# Promoting the creation of R&D intentions in primary healthcare measured by a validated instrument

**DOI:** 10.1186/s12961-019-0513-3

**Published:** 2019-12-30

**Authors:** Helena Morténius, Amir Baigi

**Affiliations:** 1 Department of Health Care, Region Halland, Box 517, 301 80 Halmstad, Sweden; 2 Department of Primary Health Care, The Sahlgrenska Academy, University of Gothenburg, Gothenburg, Sweden; 3 Department of Research and Development, Region Halland, Halmstad, Sweden

**Keywords:** Change in work practice, interest in R&D, primary healthcare, research and development, strategic communication, validated instrument

## Abstract

**Background:**

Primary healthcare has a long and successful patient care history in Sweden. Nevertheless, a research-oriented attitude has been more or less absent in this context. In society today, access to information has significantly influenced the nature of patients’ demand for up-to-date healthcare. A prerequisite for this new demand is health professionals who are interested in novel ways of thinking and view a change of work practices as necessary. One way to achieve this goal is by means of strategic communication, which is a relatively new interdisciplinary field. The aim of this study was to analyse the role of strategic communication in the creation of intentions in Research and Development (R&D) among primary healthcare staff as measured by a validated instrument.

**Methods:**

An intervention study on staff was performed. A 15-item questionnaire was validated and implemented. All primary healthcare staff from the southwestern Swedish province of Halland were included. In total, 846 employees (70%) agreed to participate in the measurements. After 12 years, 352 individuals who had participated in the intervention and remained in the organisation were identified and followed up. The intervention comprised established communication channels. The measurements were performed after 7 and 12 years. A questionnaire was designed for this purpose. The questions were validated by a factor analysis, and the degree of reliability was measured with Cronbach’s alpha coefficient. χ^2^ and Fisher’s exact tests were used as statistical tests in comparisons.

**Results:**

Factor analysis identified five pure factors (most Cronbach’s alpha > 0.70). Strategic communication contributed to a significant improvement in the staff members’ interest in R&D and willingness to change in both the short (*P* < 0.05) and long (*P* < 0.05) term. The positive attitude was stable over time.

**Conclusions:**

Strategic communication seems to be a significant tool for creating a stable positive attitude towards R&D in the primary healthcare context. The creation of a positive attitude towards a scientific approach is a relevant finding that deserves special attention in a context as complex as healthcare. Using a validated instrument seems to contribute to pure results in this case.

## Background

The healthcare context is often described as complex and as having several layers, including individuals (multiple healthcare professionals, non-clinical staff, patients) and organisations (primary healthcare infrastructure, technology, computerised information systems, delivery of treatments to patients, culture and working practices), but the concept of complexity is subjective and depends on the context [1]. The term ‘complex’ is frequently employed in the scientific literature to describe tasks or systems ranging from complicated to almost impossible to manage, suggesting that many different measurements would be required to capture all intuitive ideas about complexity [2]. New information and communication technologies have changed the world. Over the past two decades, Internet use has increased and, currently, almost everyone has the opportunity to be ‘connected’. In current society, access to this flow of information through various digital platforms has significantly influenced patient demand for healthcare. The one-sided monologue from a doctor and nurse to a patient has fundamentally changed due to patients’ ability to perform online research; the monologue has become a dialogue. The current situation requires a better understanding of how staff have adapted to patients’ recent research abilities. New technological developments and the critical approach used by patients have led to the emergence of new healthcare areas and services. Patients increasingly search for medical information on the Internet before contacting care services. Users must be able to filter the rapidly expanding flow of information, particularly information that is not medically relevant. In turn, healthcare staff have become increasingly aware of the need to apply a critical mode of thinking and to closely examine the sources of evidence-based information [3, 4]. Healthcare organisations are complex and comprise many different professional categories and operational areas in addition to several managerial levels; thus, gaps frequently arise between everyday practices and theoretical ways of thinking [5]. For example, practitioners lack sufficient ability and ambition to implement evidence-based interventions [6, 7]. This and similar situations have led to the hiring of experts to promote awareness, knowledge, skills and self-efficacy and to create motivation among the staff to adopt evidence-based interventions [8–10]. In clinical practice, complexity is considered an important factor in patient safety and quality care [11, 12]. The existence of multiple healthcare processes in which several factors and professional categories exert varying degrees of influence on availability and practice lead to unpredictable outcomes [13]. The current situation requires healthcare professionals to keep abreast of changes in and updates to scientific development in the field. Therefore, creating awareness of research and development (R&D) within the organisation is necessary to promote and strengthen a positive attitude towards change for the long-term benefit of patients. The use of strategic communication among primary healthcare staff is one way to achieve such a change and has been studied in the past [14–18].

However, a readiness to change is required to establish an attitude that is conducive to new thoughts and ideas. An organisation’s readiness to change (ORC) is defined as “*the extent to which organisational members are both psychologically and behaviourally prepared to implement change*” [19]. The chances of successful implementation are greatly improved when there is preparedness for constant change, thus avoiding a waste of resources [20].

The translation of research into practice has been neglected [21]. In this respect, an ORC can serve as a facilitator of efficient knowledge translation (KT). In the healthcare context, KT is the process of transferring knowledge from where it was created to where it can be refined and used in clinical practice and patient care [22]. An organisation characterised by systematic readiness for change is prepared to assess and anticipate the consequences of such a change. An important aspect of KT is the identification of methods for assessing an ORC [23]. Such a method could be strategic communication, which can promote the organisation of and reduce the gap between research and practice. Strategic communication has been defined as “*the purposeful use of communication by an organisation to fulfil its mission*” [24].

### Strategic communication

Strategic communication is a relatively new interdisciplinary field developed in the early 2000s [25, 26]. The main areas of application include media and communication, sociology, political science and psychology. Strategic communication is based on several theories derived from the abovementioned disciplines, e.g. the social learning theory, information process theory and diffusion of innovation theory [27–29]. Communication is optimal when the context in question is open to new ideas either prior to or during the consolidation process [30, 31]. The communication targets are achieved using established communication channels targeting specific groups by processes that provide objectively measurable results [32]. Strategic communication can be used as a tool in an organisation to create new attitudes towards willingness to change [14]. In a complex context, such as healthcare organisations, a change in attitude does not occur smoothly, and significant barriers are a natural part of the process [14]. Furthermore, perseverance and a structured approach are necessary to sustain the changed staff attitudes as the acceptance process is gradual (implicit attitudes) among practitioners [33]. Strategic communication acts through different channels but in the same direction to achieve the long-term goal. Evaluation of communication efforts has historically been narrow, measuring the number of visitors to events or clicks on a website, yet an integrated model for evaluating strategic communication that captures inputs, activities, outputs and outcomes to impact is preferred [34]. Validated instruments exist that measure components of personal health and staff ability to change work practices in general. Specifically, there is no validated instrument to measure the staff’s intention to engage with R&D, meaning their knowledge and interest as well as their creation of new thinking and willingness to change working practices. Establishing such instruments with regards to content and construct validity is therefore desirable in this subject.

### Aim

The aim of this study was to analyse the role of strategic communication via direct and indirect communication channels in the creation of interest in R&D and the willingness of primary healthcare staff to change their work practices as measured by a validated instrument*.* The purpose was to see if the validation of instruments could give a clearer result compared to the previous studies where the impact of single questions was examined.

## Methods

### Design and settings

#### Primary healthcare organisation

The Swedish primary healthcare organisation has a long tradition of patient care. Nevertheless, the level of research orientation within the organisation is fairly low, which has created difficulties in generating a demand for research in the field [35]. Historically, research has not been prioritised in primary care, leading to a lack of interest [36, 37], which can be linked to the absence of the following two important factors: a supportive infrastructure and a facilitative research culture [38]. Thus, investigating how the combination of behavioural factors, contexts, organisations and individuals influences the willingness to change work practices among primary healthcare staff is important [39, 40].

#### Primary healthcare context

Primary healthcare constitutes the foundation of health and medical care for the population. Its areas of responsibility include medical treatment, health promotion, disease prevention, rehabilitation and nursing care [41]. In 1996, new legislation stipulating the establishment of R&D units was introduced to enhance scientific competence and willingness to engage in research [42]. This new legislation facilitated research outside university hospitals. As a result, R&D units were established in primary healthcare organisations in Sweden. In general, these units are financed by the public sector.

### Study design

We conducted an intervention study with a 12-year follow up.

#### Population and intervention tool

The size of the sample was determined based on the research team members’ empirical assumption that the overall influence of communication on changes in attitude over time would be approximately 40%. Given an expected hypothetical effect of at least 30% (beta error = 0.20; power = 0.80) and a significance level of 0.05, approximately 172 individuals were required in the study cohort to demonstrate a probable statistically significant improvement. A paired sample of 352 individuals, including the follow-up of the cohort, should therefore be satisfactory to draw conclusions based on the results obtained [43].

The intervention comprised all primary healthcare staff in the province of Halland in south-western Sweden; in total, 846 employees (70%) agreed to participate in the measurements. After 12 years, all 352 individuals who had participated in the intervention and remained in the organisation were identified and followed up. Strategic communication served as a tool for creating knowledge of R&D and a springboard for generating interest, new thoughts and a willingness to change existing work practices. All communication channels that exerted direct or indirect influence on staff were included. The communication was carried out by the R&D staff, with the communication expert as a team leader. The measurements were performed after 7 years (short term) and 12 years (long term). Questionnaires marked with a unique number were distributed to the home addresses of staff together with a cover letter and a prepaid response envelope. Participants were guaranteed confidentiality and the ethical aspects of the study were described.

#### Intervention process

**Fig. 1:**
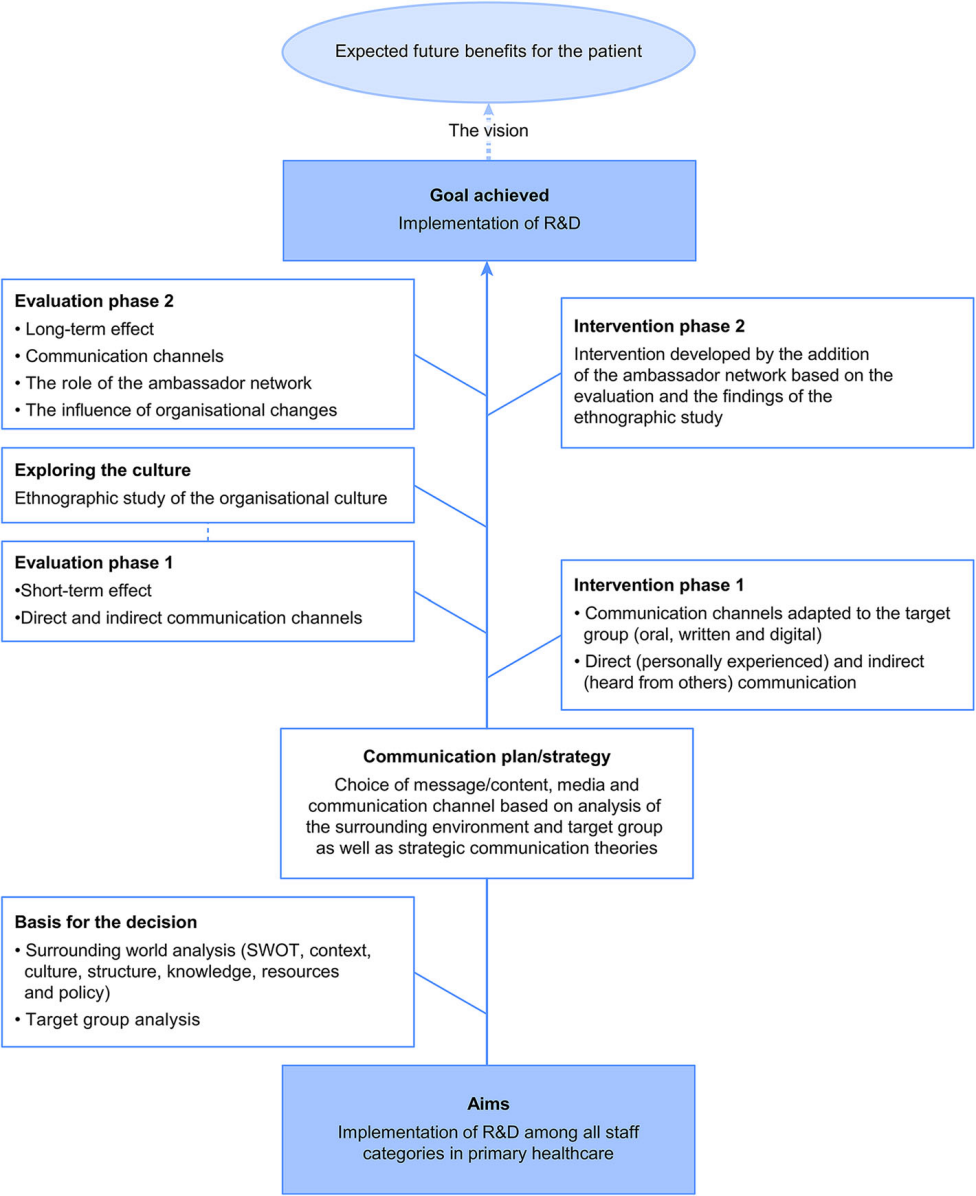
The planning, intervention, follow-up and evaluation of R&D implementation in healthcare

The design comprised established communication channels and role models (Fig. 1). The strategic communication employed in the staff cohort was influenced by a theoretical framework in terms of both the design of the communication plan and the performance of the intervention [43]. First, a surrounding world analysis was performed to obtain information about the current state of R&D and scientific resources; then, this information was used to generate the communication plan, which served as the basis of the intervention. The surrounding world analysis subsequently formed an underlying view of the current state of R&D in the organisation [44]. The results revealed a low level of R&D activity among the staff, highlighting the issue of systematic resource allocation within the activities. Subsequently, the communication plan was operationalised to reach the goal of raising the scientific competence of all employees in the primary care area. The plan consisted of creating a preparatory scientific way of thinking with the purpose of preparing a scientific mindset for awareness and interest in R&D, innovation and the willingness to change work practices.

Three established communication channels were used. Oral channels served as a ‘popular-science’ style method of diffusing information about research projects in the organisation. Research seminars and annual research days are examples of oral channels, while the written channels include an R&D bulletin, research reports and popular science reports. This concept is based on communication theories involving the creation of interest, dissemination of knowledge and evaluation of results. The language used in the R&D bulletin adopted a ‘popular science’ style and aimed to create an interest in research, development and critical thinking. The R&D bulletin appeared four times per year, and this regularity was important for facilitating the dissemination of information about ongoing and future R&D activities, such as lectures and research courses, in addition to presenting reports, images and news forms, e.g. annual research conferences. The R&D bulletin also presented and profiled those members of the organisation who were the most active in R&D, thus contributing to the shaping of role models [27]. Instead of being sent to each staff member’s home, copies of the R&D bulletin were placed in common locations, such as the coffee room, to increase exposure and to encourage its contents to become a topic of conversation. Distributing the R&D bulletin to only unit managers was deemed risky, as the managers might give the bulletin only to certain selected staff members, thus functioning as gate-keepers [29]. The digital channels were initially straightforward but gradually developed into active sites for the distribution of news and in-depth texts, complementing the written and oral channels by regularly providing topical information about R&D in primary healthcare. Information was disseminated through these direct channels, involving active participation/use, as well as through indirect exposure channels, including by staff members or managers involved in R&D activities, who communicated information to their colleagues. Efforts were made to ensure that all three channels satisfied the R&D information needs of all staff members. It was expected that, in the long term, interactions among the channels’ various information activities would promote the intention of staff members to engage in R&D. As R&D was a new concept in the organisation, the focus was on information dissemination to and acceptance of its importance by both the whole organisation and its members. The communication channels’ activities were adapted to different target groups in the context of the study (Fig. 1) [29]. From the beginning, the entire communication process was established in a scientific manner to allow subsequent evaluation.

#### Measurement instrument

A questionnaire was employed to measure the changes in attitude during the intervention period. The questionnaire comprised 16 items pertaining to the channels’ influence on the awareness of R&D (7 direct channels; 9 indirect channels), including 15 items used for validation purposes. The item ‘Participated in an R&D course (D)’ did not contribute to factor formation and was, therefore, excluded from the validation process. These items have been used as independent variables in earlier studies [14]; thus, their interaction effect was not considered (Table 1).

**Table 1:** The 15-item instrument and distribution of the participants’ responses on two occasions (*n* = 352)

		*n*	
		Occasion I	Occasion II
Regarding one’s own initiative (direct)			
1	Read a popular science report	198	184
2	Read the research bulletin	270	252
3	Read information on the Intranet	211	206
4	Read information on the Internet	152	156
5	Attended a scientific seminar	190	182
6	Attended a research conference	211	205
Heard about somebody who had (indirect)			
7	Described an R&D project	246	228
8	Read a popular science report	163	164
9	Read the research bulletin	172	182
10	Read information on the Intranet	163	154
11	Read information on the Internet	141	142
12	Attended a scientific seminar	154	147
13	Attended an annual research conference	166	159
14	Participated in an R&D course	174	161
15	Was informed about R&D at the management level	162	152

To determine the staff’s newly created intentions through strategic communication after the start of the R&D Unit, the following questions emerged as the focus:

- Has the R&D information in your organisation led to you…
- Developing a new way of thinking and ideas?
- Changing or intending to change your work practices?

### Validation process

The process of validating the questionnaire comprised three steps, namely item construction, face and content validity, and construct validity.

#### Step I: Item construction

A questionnaire was constructed on the basis of a literature review and the knowledge of the research team. The team consisted of a primary healthcare communication strategist (main author), a general practitioner (primary healthcare physician), an expert in strategic communication, a healthcare expert (nurse) and a biostatistician (public health), all of whom worked together in developing and scrutinising the items. The design of the items and their relevance were discussed, and adjustments were made in a continuous process of questioning the items until the group members’ understanding had reached a saturation level.

#### Step II: Face and content validity

Once the questionnaire acquired a preliminary structure, it was distributed among the members of a pilot group. The pilot study was carried out in two steps. First, employees and contract employees (*n* = 20) were requested to read and reflect on the items and provide suggestions for improvement. The reflections were based on an exploration of the interpretation of the items and the relevance of the response alternatives and were implemented by an assessment of the readability and comprehensibility of the words and sentences. The second step was performed after the questionnaire had been amended and involved a strategic selection covering an even geographical distribution of primary healthcare areas in the county (*n* = 50) [43]. Subsequently, the staff members completed and reflected upon the questionnaire similarly to the participants of the pilot study. The revised questionnaire was further discussed in the pilot group and subjected to scrutiny by the expert group [45]; the questionnaire was then distributed to all primary healthcare staff members in the county who participated in the intervention.

#### Step III: Construct validity

##### Factor analysis

To obtain the pure factors, the construct validity of the questions was measured using an explorative factor analysis [46–49]. All items were included in the factor analysis and were studied in exactly the same way on both measurement occasions, which were separated by a 5-year interval. The population size (*n* > 150) in the study was optimal for implementing this technology, as several high loading variables were identified [50, 51] and the recommended ratio of participants to items, i.e. 10 cases for each item in a factor analysis, was achieved [52]. To consider the suitability of the factor analysis, Bartlett’s test of sphericity was used [53]. The Kaiser–Meyer–Olkin test was performed to measure the adequacy of the sampling [54, 55]; a Kaiser–Meyer–Olkin value greater than 0.6 was deemed optimal for carrying out a factor analysis [50]. A principal component analysis was utilised as the factor extraction technique in which a satisfactory solution was based on the criterion that only factors with an Eigenvalue greater than or equal to 1.0 were included in further analysis [48, 50]. Factor loadings ≥ 0.30, including a total variance > 50%, were considered meaningful [48, 56]. The main approach to the rotation was based on the mean of the orthogonal factor solution with the Varimax rotation method. The homogeneity reliability was measured using Cronbach’s alpha coefficient, and a value greater than or equal to 0.70 was considered appropriate [48].

### Statistical analysis

#### Measurement of impact

The impact of the factors on R&D interest, the new way of thinking and willingness to change work practices was measured using χ^2^ and Fisher’s exact tests. The significance level was set at 0.05. All tests were double-sided.

#### Power of the study

The sample size was based on the anticipated impact of strategic communication on interest in R&D and willingness to change work practices. Both the short- and long-term effects were considered. As we were unable to find a similar study design in the published literature that could provide an indication of a preliminary effect size, the research team decided that the intervention should encompass all primary healthcare staff in the region.

#### Patient and public involvement

The study design did not include patients. The staff were informed of the importance of the survey for the primary care organisation in the long term. They did not participate in planning the study design. Furthermore, the research team formed a multidisciplinary group employed in the primary care organisation.

## Results

### Construct validity

A factor analysis was employed to reduce the number of items, and this process was performed identically on the two measurement occasions. The exception was item *g*, which did not form a factor and thus was excluded from the second occasion. In total, 15 items formed the following five pure factors: Seminar/conference, Bulletin/report, Intranet, Committed staff/manager and Internet. The item ‘Participated in an R&D course him/herself’ did not contribute to the formation of these five factors. The total variance was > 0.50. Similarly, the communality values were > 0.50, except for the item ‘Participated in an R&D course (ID)’, which had a somewhat lower value. There was a strong correlation among all five factors (> 0.56), and the values were identical on the two measurement occasions. The degree of homogeneity among the factors satisfied the requirement for an alpha coefficient > 0.70, except for the factor ‘Committed staff/manager’, which had lower values on both occasions, i.e. 0.52. The overall alpha coefficients were 0.78 and 0.79 (Table 2).

**Table 2:** Construct validity (factor analysis) and homogeneity (Cronbach’s alpha coefficient). Utilisation of the 15-item instrument

Items	Total variance %		Communalities		Seminar/conference		Bulletin/report		Intranet		Committed staff/manager		Internet	
Measurements I and II	I	II	I	II	I	II	I	II	I	II	I	II	I	II
	64.6	58.7												
Attended a scientific seminar (ID)			0.71	0.66	0.79	0.68								
Attended a research conference (ID)			0.75	0.61	0.78	0.64								
Attended a scientific seminar (D)			0.67	0.57	0.75	0.71								
Attended a research conference (D)			0.56	0.63	0.73	0.76								
Cronbach’s alpha:					0.82	0.76								
	55.2	55.1												
Read the research bulletin (D)			0.67	0.58			0.76	0.66						
Read a popular science report (ID)			0.67	0.69			0.67	0.74						
Read the research bulletin (ID)			0.54	0.55			0.67	0.64						
Read a popular science report (D)			0.50	0.64			0.63	0.73						
Cronbach’s alpha:							0.73	0.80						
	87.4	83.4												
Read information on the Internet (D)			0.86	0.70					0.90	0.82				
Read information on the Internet (ID)			0.85	0.74					0.90	0.81				
Cronbach’s alpha:									0.84	0.80				
	50.8	51.0												
Was informed about R&D at the management level (ID)			0.52	0.64							0.68	0.78		
Participated in an R&D course (ID)			0.68	0.44							0.62	0.57		
Described an R&D project (ID)			0.63	0.51							0.61	0.61		
Cronbach’s alpha:											0.52	0.52		
	84.1	77.8												
Read information on the Intranet (D)			0.79	0.76									0.87	0.85
Read information on the Intranet (ID)			0.78	0.68									0.86	0.67
Cronbach’s alpha:													0.81	0.71
Cronbach’s alpha (overall): Measurement I: 0.78 Measurement II: 0.79														

*D* direct communication, *ID* indirect communication

### Changing attitudes through R&D communication

The strategic communication aimed to convey the R&D message and to create an interest in R&D by the following direct and indirect communication activities: direct communication occurred when an individual carried out the activities, (D) and indirect communication occurred when someone else informed other individuals about the activities (ID).

#### Seminars and conferences

Active participation in R&D seminars and annual R&D conferences, followed by receiving information through a third party (managers and/or colleagues), were found to have significant influences on R&D interest in both the short and long term (*P* < 0.05). Seminars and conferences were strongly associated with staff members’ new ways of thinking and willingness to change work practices in both the short and long term (*P* < 0.05).

#### Committed staff/managers

General information about R&D from management, followed by specific information from those who participated in R&D education or an R&D project, also contributed to interest in R&D in the short and long term (*P* < 0.05). Similar to seminars and conferences, a strong association was found between a new way of thinking and willingness to change work practices in the short and long term (*P* < 0.05).

#### R&D bulletin/reports

The two most frequently employed communication channels, i.e. the R&D bulletin and popular science reports (D/ID), were the greatest contributors to creating R&D interest in the short and long term. These channels of communication also demonstrated a significant long-term association with a new way of thinking and willingness to change work practices (*P* < 0.05).

#### Websites

The digital communication activities demonstrated a partially positive influence on the staff members’ R&D interest in the long term (*P* < 0.05) (Table 3).

**Table 3:** Association between the communication channels and the creation of R&D intentions among healthcare staff

	Seminar/conference		Committed staff/manager		Bulletin/report		Internet		Intranet	
	n_1_/N_1_ (%)	*P*	n_1_/N_1_ (%)	*P*	n_1_/N_1_ (%)	*P*	n_1_/N_1_ (%)	*P*	n_1_/N_1_ (%)	*P*
Measurement I:										
R&D interest	154/240(64)	< 0.001	191/227(84)	< 0.001	267/296(90)	< 0.001	13/159(8)	NS	116/218(53)	NS
New way of thinking	109/140(78)	< 0.001	129/197(65)	< 0.001	143/228 (63)	NS	4/11 (36)	NS	60/98 (61)	NS
Willingness to change	48/65 (74)	< 0.001	64/72 (89)	< 0.023	66/74 (89)	NS	3/46 (7)	NS	28/59 (48)	NS
	n_2_/N_2_ (%)	*P*	n_2_/N_2_ (%)	*P*	n_2_/N_2_ (%)	*P*	n_2_/N_2_ (%)	*P*	n_2_/N_2_ (%)	*P*
Measurement II:										
R&D interest	161/229 (70)	< 0.001	210/248 (85)	< 0.001	241/279 (86)	< 0.001	29/160 (18)	NS	132/215 (61)	0.001
New way of thinking	111/144 (76)	< 0.001	129/143 (69)	< 0.001	150/166 (90)	0.002	15/91 (17)	NS	77/125 (62)	NS
Willingness to change	50/69 (73)	< 0.021	66/73 (90)	0.008	67/75 (89)	0.049	8/54 (15)	NS	39/68 (57)	NS

N_1_: Participated in the intervention and replied to the questionnaire

N_2_: Participated in the intervention, remained in the organisation and replied to the questionnaire

n_1_: Staff who changed the attitudes in the measurement I

n_2_: Staff who changed/retained the attitudes in the measurement II

χ^2^ and Fisher’s exact tests were used

*NS* not statistically significant

## Discussion

### Summary

Strategic communication contributed to a significant improvement in R&D interest and was largely associated with new ways of thinking and willingness to change work practices among primary healthcare staff members. The positive attitude was stable over time. The validation of the instrument provided a new perspective compared to that in previous studies as the role of communication in the intervention process was highly strengthened.

### Method issues

#### Study design

In conducting a prospective study, baseline information is an important methodological factor. The only document available was a background report that established that no R&D culture existed within Region Halland primary healthcare [44]. Therefore, this report was complemented by the researcher’s experience based on many years of employment in this context. As baseline data were lacking, the questionnaire items were constructed, and the follow-up questions were designed to allow the participants to state whether their intention to engage in R&D had been directly influenced by the strategic communication. The intervention included all primary healthcare staff in Region Halland. No controls were recruited due to the disparity among existing national R&D units and the lack of uniformity of information provision in these organisations. Furthermore, the selection of controls would not meet the inclusion criteria [57, 58]. Since the study utilised a prospective 12-year design, following up on the long-term influence of the communication was feasible, thus providing a good overview of its impact. The intervention study comprised all professional categories, which was significant since this is an important factor in the creation of a culture across the whole healthcare chain characterised by the intention to engage in R&D [43].

#### Validation of the instrument

A validated questionnaire is preferable for research purposes [57]. As no such validated questionnaire could be found, our self-designed questionnaire underwent a validation process. The items pertaining to the communication channels were validated before and during the follow-up (occasion I) and during the final evaluation (occasion II), yielding identical factors. This finding should be considered a satisfactory validation of the items’ categorical scale. The validation would have been more straightforward if the questions had a continuous distribution since factor analysis is based on average estimation. The number of validated instruments within the communication and implementation science field is limited. More specifically, there are few validated items regarding the role of communication channels in changing attitudes. By applying a four-phase process, our research team constructed, operationalised, implemented and validated 15 items that could be combined to measure five different aspects of communication activities. The items measuring the staff members’ direct participation and indirect exposure to the communication channels covered the oral, written and digital channels and their influence on changes in attitudes in the long and short term. In previous studies, the associations of these 15 items with related items were not investigated; thus, their interaction was not considered [14] and any synergy effects within the domains could not be observed. The design of the items was improved by discussions and reflections among the interdisciplinary interprofessional research group [43] and considering the views of the pilot group members about how to formulate the basic pedagogical structure of the instrument in a logical way. The resulting adjustments contributed to a relatively high factor correlation within each domain with good to excellent communalities with a relatively good value for Cronbach’s alpha [48]. However, the alpha value of the domain ‘Committed staff/manager’ was somewhat lower, which to some degree can be explained by the small number of items in this domain. This type of analysis is especially suitable for items with a rating scale [46, 48]. As the optimal utilisation of the test requires numerical (parametric) data, the factors were checked by Spearman’s correlation, which indicated equal correlations between the items.

### General principle

It is widely recognised that evaluations of a self-constructed instrument likely contribute to a conflict of interest rather than describe the reality of the study context [59]. Nevertheless, this concern can serve as motivation to maintain an objective stance in planning, implementing and analysing a study. The same principle applies to the researchers’ association with the R&D unit. However, such challenges were reduced due to the interdisciplinary composition of the team and the fact that its members are practitioners in the areas of medicine, biostatistics, nursing and strategic communication.

### Discussion of the results

The individual impact of the communication activities on attitude towards R&D and R&D intention has been previously investigated [43], whereas their interaction within a group (internal synergy) has not been considered. A factor analysis was employed to elucidate the covariation between the direct and indirect channels and the impact on the change in the staff members’ attitudes. This approach is consistent with the current interpretation of the interaction between direct and indirect channels and the way in which they are generally used in social media, where the views of significant others are prioritised over those of authorities, researchers and other external actors. Thus, communication in the organisation was provided by opinion leaders and role models who, in turn, conveyed the R&D messages to their colleagues. An innovation process requires time, as some staff members assimilate the innovation less quickly than others [30]. Therefore, the primary aim of strategic communication is to first create an interest in research towards a new way of thinking that would eventually result in the willingness to change work practices in the organisation [14]. As this concept is relatively new to the context [60], great emphasis was placed on diffusing knowledge and gaining acceptance of R&D. An advantage of this strategy is that it contributed to R&D receiving increasing attention, curiosity being stimulated and R&D becoming a topic of discussion [32, 61], potentially explaining why the staff members started to attend scientific seminars and research conferences. As long-term behavioural change is, in most cases, a time-consuming process [5], it is especially important to be able to establish a continued high level of R&D intention on a longitudinal basis [16], which was also consistent with the primary aim of the intervention. By adapting the message to the target group and using the language of popular science, larger groups of staff were reached. Furthermore, it is reasonable to assume that the pedagogical platform contributed to an environment that was easy to understand and less formal, paving the way for new thinking and willingness to change. Other contributory factors were the synergistic effects among the various communication activities and the pedagogical platform, followed by the long-term design [16]. An advantage of long-term ambition is that it creates stability in attitudes towards change, thereby paving the way for an organisational culture that is open to change [62]. However, during a long-term intervention, hidden confounders may act as a hindrance and exert a negative impact on the outcome. Research has demonstrated that the longer a person has been employed in the same workplace, the lower their willingness to utilise research findings [63]. In the present context, a potential confounder was the organisational culture [64]. This hypothesis is consistent with Morténius et al. [16], who reported a negative correlation between length of employment and willingness to change work practices. Nevertheless, the activities of the strategic communication channels contributed to a shift in paradigm within Region Halland, where an interest in R&D and a willingness to change were observed among all staff members. This outcome is consistent with one of the great challenges in healthcare highlighted by WHO, namely, bridging the research-practice gap [65]. In view of the low implementation level of research results within healthcare, it is vital to create R&D intentions and willingness to change [66], despite access to established platforms such as the i-PARIHS framework [67]. Due to the complexity of the healthcare organisation and varying knowledge levels, a change in staff members’ attitudes towards R&D could promote greater equality in a longer perspective in terms of readiness to change, first by influencing the organisational environment in a positive way, and second as a contributory factor in the creation of ORC.

### Main issues

A scientific mode of thinking and intention to implement research results in healthcare are necessary. In the current information society, patients place increased demands on healthcare staff to be up-to-date on the most recent research, requiring encounters with patients to be characterised by a scientific stance. A long-term investment in research and innovation is essential for meeting future challenges in healthcare. However, in the practical context, research and innovation should occur in cooperation with different actors such as industry, pharmaceutical companies and universities [68]. Thus, the implementation strategy in a complex system, such as healthcare, must be adapted to the target group’s level of knowledge. Several established frameworks, such as Cynefin [69] and i-PARIHS [67], are based on similar concepts. Strategic thinking is an important factor that includes many different aspects, such as knowledge about the organisational structure, readiness to change, climate, culture and the acceptance levels of different target groups. Therefore, it is essential to assume a long-term view of willingness to change, which is the basis of a shift in paradigm. However, the complexity requires an analytical approach and the ability to interpret several interactive dimensions in the organisation. Gaining acceptance of new thinking and willingness to change is an intricate process in a complex organisation and can easily be jeopardised by simple solutions, resulting in chaos in the system [69]. In the i-PARIHS framework, emphasis is placed on the use of facilitators with different levels of experience (novice, experienced and expert) [67], which is an aspect that should be further developed in healthcare. During the process of the implementation of a strategy to change attitudes, R&D organisations should be considered natural bridge builders, i.e. experts, due to their 'strong links with both academic institutions and healthcare organisations', in addition to their knowledge about building networks in a specific context [43, 67]. However, the involvement of facilitators should be carefully structured to ensure that the theoretical background and practical implementations are combined in an optimal way [70].

### Operative role of communication

From a wider perspective, it is likely that the synergistic effect of the communication channels will change. The new wave of multivariation in communication gradually erases traditional boundaries. For example, social media facilitates interactions, dialogues, instant feedback and opportunities to obtain a quick overview of the surrounding world. Thus, an organisation can create new, converging forms of content and utilisation. Despite the strength of strategic communication that can be adapted to the context and communication requirements of different target groups, the new wave of communication via social media places demands on its design and content to provide an optimal effect, which may lead healthcare organisations to considerably revise their communication channels in the near future, thus contributing to additional ways of communication and enhancing the importance of the message rather than the choice of channel. This does not apply to communication through traditional or social media but concerns the choice of the most suitable medium that matches the aim of the message [71]. For the optimal utilisation of strategic communication in each unique organisation, a communication strategist has to be an integral part of the context. This person should be an expert who ensures that the practical adaptation targets relevant target groups in the organisation by applying a theoretical approach [43, 72].

Furthermore, this study provides a valuable evaluation of an instrument to assess the implementation of changing attitudes in a primary healthcare organisation. The study draws attention to contextual and staff aspects for future innovative planning in the healthcare sector and, as a consequence, for patient benefit in the long term. The creation of an organisational culture with a willingness to change work practices is also valuable for implementing new guidelines and research findings in the organisation. Furthermore, the study design and methodology can be used for educational purposes in the future.

## Conclusions

Strategic communication seems to be an important tool for changing attitudes in the primary healthcare context in both the short and long term. Our finding that a positive attitude towards R&D was created in a complex context, such as healthcare, and could be measured by a validated instrument merits special attention, particularly in view of the patient perspective.

## Data Availability

Data are available upon reasonable request from the corresponding author.

## Abbreviations

KT: knowledge translation
ORC: organisation’s readiness to change
R&D: research and development
